# Spaces of ranked tree-child networks

**DOI:** 10.1007/s00285-025-02265-2

**Published:** 2025-09-02

**Authors:** Vincent Moulton, Andreas Spillner

**Affiliations:** 1 School of Computing Sciences, University of East Anglia, Norwich, UK; 2 Merseburg University of Applied Sciences, Merseburg, Germany

**Keywords:** Ranked phylogenetic network, Equidistant network, Nearest neighbor interchange, CAT(0)-orthant space

## Abstract

Ranked tree-child networks are a recently introduced class of rooted phylogenetic networks in which the evolutionary events represented by the network are ordered so as to respect the flow of time. This class includes the well-studied ranked phylogenetic trees (also known as ranked genealogies). An important problem in phylogenetic analysis is to define distances between phylogenetic trees and networks in order to systematically compare them. Various distances have been defined on ranked binary phylogenetic trees, but very little is known about comparing ranked tree-child networks. In this paper, we introduce an approach to compare binary ranked tree-child networks on the same leaf set that is based on a new encoding of such networks that is given in terms of a certain partially ordered set. This allows us to define two new spaces of ranked binary tree-child networks. The first space can be considered as a generalization of the recently introduced space of ranked binary phylogenetic trees whose distance is defined in terms of ranked nearest neighbor interchange moves. The second space is a continuous space that captures all equidistant tree-child networks and generalizes the space of ultrametric trees. In particular, we show that this continuous space is a so-called CAT(0)-orthant space which, for example, implies that the distance between two equidistant tree-child networks can be efficiently computed.

## Introduction

Rooted phylogenetic networks are essentially directed acyclic graphs, whose leaf sets correspond to a set of species. They are commonly used to represent evolutionary histories in which reticulate events have occurred due to processes such as hybridization and lateral gene transfer. Various classes of rooted phylogenetic networks have been defined, including the extensively studied class of so-called *tree-child networks* introduced by Cardona et al. (2008) (see e.g. Kong et al. (2022) for a review). Recently, the class of (binary) *ranked tree-child networks (RTCNs)* was introduced by Bienvenu et al. (2022), which have been further studied by Caraceni et al. (2022) and Fuchs et al. (2024). As their name suggests, these are a special type of tree-child network that are endowed with additional information which allows the evolutionary events represented by the network to be arranged consistently along a time line. RTCNs generalize *ranked phylogenetic trees* (also called *ranked genealogies*), structures that can be used to study evolutionary dynamics (see e.g. Kim et al. (2020) and the references therein).

**Fig. 1:**
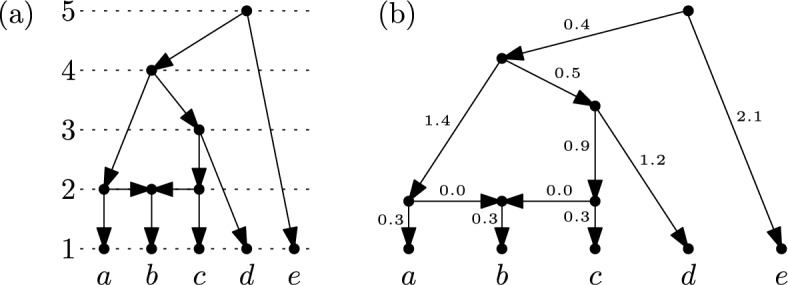
(**a**) A binary RTCN on the set $X = \{a,b,c,d,e\}$. Each dotted horizontal line corresponds to vertices that have the same rank. (**b**) An ETCN on $X$ obtained by assigning suitable weights to the arcs of the RTCN in (**a**)

Informally (see Sect. 2 and Sect. 6 for full definitions), a binary RTCN is a binary rooted phylogenetic network with leaf set *X* having the following additional restrictions: (i) every vertex that is not a leaf must be the tail of some arc whose head has no other in-coming arcs, (ii) vertices are assigned ranks from the set $\{1,\dots ,|X|\}$ such that the tail of an arc never has a smaller rank than the head, and (iii) the head and the tail of an arc have the same rank if and only if the head has two in-coming arcs. Condition (i) restricts the topology of the network to that of a tree-child network, Condition (ii) arranges the vertices along a time line, and Condition (iii) captures the idea that the network represents a sequence of two types of evolutionary events, namely branchings and reticulations (cf. (Bienvenu et al. (2022), Sec. 1.2)). In Fig. 1(a) we give an example of a binary RTCN. In addition, by assigning non-negative weights to the arcs of an RTCN that are consistent with the ranks of the vertices (in particular, vertices having the same rank also have the same distance from the root) we obtain an *equidistant* tree-child network (ETCN). An example of an ETCN is given in Fig. 1(b). Note that, if every vertex in an ETCN has at most one in-coming arc, then it is also referred to in the literature as an *ultrametric tree* (cf. e.g. (Steel (2016), p.114)).

Comparing phylogenetic trees and networks is an important problem in phylogenetics which has been studied for some time, and various distances have been defined on trees and networks (see e.g. Cardona et al. (2008); Huber et al. (2016); Janssen et al. (2018); Kuhner and Yamato (2015); Nakhleh (2009); Pons et al. (2019); Smith (2022)), including ranked phylogenetic trees (Kim et al. 2020). Thus it is a natural question to ask for ways to compare different RTCNs on the same leaf set with each other, and, similarly, different ETCNs on the same leaf set. In this paper, we shall present some new distances for such networks and consider some properties of the resulting spaces. We define our distances by introducing a way to encode binary RTCNs on a fixed leaf set $X$ in terms of a certain partially ordered set (or poset). As we shall see in Sect. 3, as well as encoding binary RTCNs, this poset has some attractive mathematical properties, including the fact that it generalizes the well-known poset of partitions of the set $X$, a poset that captures the set of all binary ranked trees with leaf set $X$ (see e.g. (Huber et al. (2024), Sec. 4.2)).

Using our new encoding, in Sect. 4 we provide a generalization of the Robinson-Foulds distance on rooted phylogenetic trees, and also define a generalization of the ranked *nearest neighbor interchange (rNNI)* distance on binary ranked trees introduced by Gavryushkin et al. (2018) to all binary RTCNs, thus providing a way to compare binary RTCNs. In addition, in Sect. 6 we define a continuous metric space of ETCNs whose definition relies on some special properties of the poset mentioned above. More specifically, we show that this space is a so-called *CAT(0)-orthant space*, which implies that the distance between any two ETCNs can be computed efficiently. Note that Billera et al. (2001) presented a similar approach to compare unrooted edge-weighted phylogenetic trees, and that our space of ETCNs generalizes the more recently introduced spaces of ultrametric trees (Gavryushkin and Drummond 2016) and equidistant cactuses (Huber et al. 2024).

We now describe the contents of the rest of this paper. After formally defining binary RTCNs in Sect. 2, we show how binary RTCNs with a fixed leaf set correspond to maximal chains of certain cluster systems (Sect. 3). Then we introduce the poset capturing all binary RTCNs, present our generalization of rNNIs and show that the discrete space of all binary RTCNs is connected under these more general rNNIs (Sect. 4). Next we describe how our poset also systematically captures certain non-binary rooted phylogenetic networks that are tree-child and have ranked vertices (Sect. 5) and use this to describe our CAT(0)-orthant space of ETCNs (Sect. 6). We conclude mentioning some possible directions for future work (Sect. 7).

## Binary ranked tree-child networks

In this section, we formally define the basic type of phylogenetic network that we consider in this paper. For the rest of this paper, $X$ will be a finite non-empty set with $n = |X| \ge 2$, which can be thought of as a set of species or taxa.

A directed graph $G=(V,E)$ consists of a finite, non-empty set $V$ of *vertices* and a set $E \subseteq V \times V$ of directed edges or *arcs*. We write $(u,v)$ for an arc that is directed from vertex $u$, the *tail* of the arc, to vertex $v$, the *head* of the arc. For a vertex $u$, the *out-degree* of $u$ is the number of arcs that have $u$ as its tail and the *in-degree* of $u$ is the number of arcs that have $u$ as its head. A *leaf* is a vertex of out-degree 0. A *directed path* in $G$ from vertex $s$ to vertex $t$ is a sequence $s=v_1,v_2,\dots ,v_k=t$ of $k \ge 1$ pairwise distinct vertices with $(v_i,v_{i+1}) \in E$ for all $1 \le i \le k-1$. Note that we allow $k=1$, which then implies that $s=t$. A directed graph $G$ is *acyclic* if it does not contain a directed path from some vertex $s$ to some vertex $t$ such that $(t,s)$ is an arc in $G$ (which would then form a directed cycle in $G$).

A *rooted phylogenetic network* $\mathscr {N} = (V,E,\rho )$ on $X$ is a directed acyclic graph $G=(V,E)$ with leaf set $X$ and a unique vertex $\rho$ of in-degree 0, called the *root* of $\mathscr {N}$. A vertex of $\mathscr {N}$ that is not a leaf is called an *interior* vertex. A vertex of $\mathscr {N}$ with in-degree at least 2 is a *hybrid vertex*. Any vertex of $\mathscr {N}$ that is not a hybrid vertex is a *tree vertex*. A rooted phylogenetic network is *binary* if the root has out-degree 2 and every other interior vertex either has in-degree 1 and out-degree 2 or in-degree 2 and out-degree 1.

**Fig. 2:**
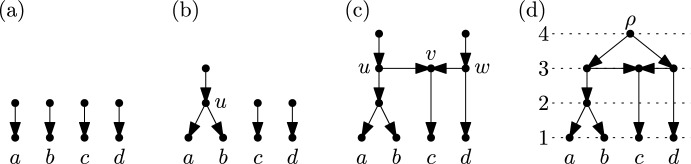
An example of the process that generates a binary RTCN on $X=\{a,b,c,d\}$. (**a**) The result of Step 1. (**b**) The result of performing (1) in Step 2. (**c**) The result of performing (2) in Step 3. (**d**) The resulting binary RTCN after Step $n=4$. Vertices of rank $i$ are drawn on the dotted horizontal line numbered $i$ $(1 \le i \le 4)$

The following definition of binary *ranked tree-child networks* (RTCN) is equivalent to the informal description of these networks given in the introduction (see (Bienvenu et al. (2022), Sec. 2.1)). More specifically, any binary RTCN on a fixed set $X$ can be obtained using a process involving $n$ steps:

- Step 1: For each $x \in X$ an arc with head $x$ is created. The tails of these arcs are pairwise distinct and form a set of $n$ vertices with in-degree 0 (see Fig. 2a).
- ${\underline{\text {Step } \ i \ (2 \le i \le n-1):}}$ Precisely one of the following modifications to the network obtained in Step $i-1$ is performed:
  1. Two vertices with in-degree 0 are selected. These two vertices are identified as a single vertex $u$ with out-degree 2. Then a new arc with head $u$ and a new vertex as its tail is added (see Fig. 2b).
  2. Three vertices $u$, $v$ and $w$ with in-degree 0 are selected. Then arcs $(u,v)$ and $(w,v)$ are added, making $v$ a hybrid vertex. Then two new arcs with head $u$ and $w$, respectively, and each with a new vertex as its tail are added (see Fig. 2c).
  After performing Step $i$ we have a network that has $n-i+1$ vertices with in-degree 0.
- ${\underline{\text {Step }n:}}$ The result of Step $n-1$ is a network with precisely two vertices with in-degree 0. These two vertices are identified as a single vertex which then forms the root $\rho$ of the resulting binary RTCN. This finishes the process of generating a binary RTCN (see Fig. 2d).

All different binary RTCNs on a fixed set $X$ arise through the choice of performing either (1) or (2) in Steps $2,\dots ,n-1$ and, subsequently, the choice of either the two vertices used in (1) or the three vertices used on (2). Note that in (2) the role of vertex $v$ is different from the roles of vertices $u$ and $w$. So, more precisely, performing (2) also involves a choice which of the three selected vertices plays the role of the vertex that becomes a hybrid vertex. If (2) is never performed in any of the Steps $2,\dots ,n-2$ the network only contains tree vertices and is called a *binary ranked tree*.

Each vertex $q$ in a binary RTCN $\mathscr {N} = (V,E,\rho )$ on $X$ has a *rank* from the set $\{1,\dots ,n\}$ associated with it that is denoted by $\text {rank}(q)$. More precisely (see Fig. 2d), we have

- $$\text {rank}(x)=1$$ for all $$x \in X$$,
- $\text {rank}(u)=i$ when (1) is performed in Step $i$ $(2 \le i \le n-1)$,
- $\text {rank}(u)=\text {rank}(v)=\text {rank}(w)=i$ when (2) is performed in Step $i$ $(2 \le i \le n-1)$, and
- $$\text {rank}(\rho ) = n$$.

These ranks correspond to an ordering of the biological events (speciation or hybridization) that led from the common ancestor at the root of the network to the elements in $X$ at the leaves. The term *tree-child* refers to the fact that in the networks generated by the process described above every interior vertex is the tail of an arc whose head is a tree-vertex. Note that tree-child networks without ranked vertices were introduced by Cardona et al. (2008) and remain an active area of research (see e.g. Cardona et al. (2019); Cardona and Zhang (2020); Fuchs et al. (2021)).

## Encoding binary ranked tree-child networks

In this section, we present a way to *encode* binary RTCNs, that is, a way to describe binary RTCNs in such a way that two RTCNs are the same if and only if they have the same description. The encoding itself is a straight-forward translation of the process described in Sect. 2 for generating a binary RTCN into the language of collections of subsets of $X$. As we will see later on, this encoding is very helpful for proving our results about RTCNs.

To formally describe the encoding, we first give some more definitions. A *cluster* on $X$ is a non-empty subset of $X$. A *cluster system* on $X$ is a non-empty collection of clusters on $X$. Given a rooted phylogenetic network $\mathscr {N}=(V,E,\rho )$ on $X$, to each vertex $v \in V$, we associate the cluster $C_{v}$ on $X$ that consists of all those $x \in X$ for which there exists a directed path in $\mathscr {N}$ from $v$ to $x$. The clusters given in this way are sometimes called the *hard-wired* clusters of the network.

Each step $i$ $(1 \le i \le n)$ in the process described in Sect. 2 can now be captured by a cluster system $\mathscr {C}_i$ on $X$ as follows:

- Step 1: $\mathscr {C}_1 = \{\{x\} : x \in X\}$. Each cluster in $\mathscr {C}_1$ consists of a single element and represents a leaf in the resulting network.
- ${\underline{\text {Step } \ i \ (2 \le i \le n-1):}}$ We already have the cluster system $\mathscr {C}_{i-1}$ which consists of the clusters $C_v$ obtained from those vertices $v$ that are the head of an arc whose tail has in-degree 0 at the end of Step $i-1$.
  - If (1) is performed in Step $i$ there must exist clusters $A$ and $B$ in $\mathscr {C}_{i-1}$ such that $C_u = A \cup B$. Then we put $\mathscr {C}_i = (\mathscr {C}_{i-1} - \{A,B\}) \cup \{C_u\}$.
  - If (2) is performed in Step $i$ there must exist clusters $A$, $B$ and $C$ in $\mathscr {C}_{i-1}$ such that $C_u = A \cup B$ and $C_w = B \cup C$. Then we put $\mathscr {C}_i = (\mathscr {C}_{i-1} - \{A,B,C\}) \cup \{C_u,C_w\}$.
  The cluster system $\mathscr {C}_i$ consists of $n-i+1$ clusters.
- ${\underline{\text {Step }n:}}$ The cluster system $\mathscr {C}_{n-1}$ consists of two clusters $A$ and $B$ such that $C_{\rho } = A \cup B = X$. We put $\mathscr {C}_n = (\mathscr {C}_{n-1} - \{A,B\}) \cup \{C_{\rho }\} = \{X\}$.

To illustrate this definition, consider again the example of generating a binary RTCN on $X=\{a,b,c,d\}$ in Fig. 2. Then we obtain the following cluster systems:

$$\begin{aligned} \mathscr {C}_1&= \{\{a\},\{b\},\{c\},\{d\}\},\\ \mathscr {C}_2&= (\mathscr {C}_1 - \{\{a\},\{b\}\}) \cup \{\{a,b\}\} = \{\{a,b\},\{c\},\{d\}\},\\ \mathscr {C}_3&= (\mathscr {C}_2 - \{\{a,b\},\{c\},\{d\}\}) \cup \{\{a,b,c\},\{c,d\}\} = \{\{a,b,c\},\{c,d\}\}, \text{ and } \\ \mathscr {C}_4&= (\mathscr {C}_3 - \{\{a,b,c\},\{c,d\}\}) \cup \{\{a,b,c,d\}\} =\{\{a,b,c,d\}\}. \end{aligned}$$

As also illustrated by this example, the cluster systems can easily be read from the resulting binary RTCN $\mathscr {N} = (V,E,\rho )$ on $X$: For $1 \le i < n$, we have

$$\begin{aligned} \mathscr {C}_i = \mathscr {C}_i(\mathscr {N}) = \{C_{v}: \text {there exists an arc} \ (u,v) \in E \ \text {with rank}(u) > i \ge \text {rank}(v)\}, \end{aligned}$$

and $$\mathscr {C}_n = \{C_{\rho }\} = \{X\}$$.

**Fig. 3:**
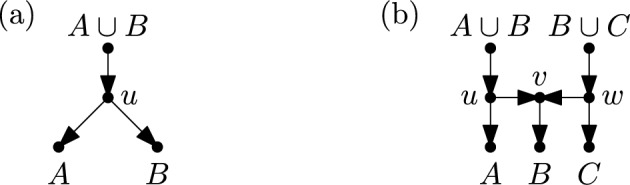
The two operations (**a**) $\vdash _{(1)}$ and (**b**) $\vdash _{(2)}$ that can be applied to a cluster system and how they are related to the process of generating binary RTCNs

To make more precise our way of encoding a binary RTCN $\mathscr {N}$ by the cluster systems

$$\begin{aligned} \mathscr {C}_1(\mathscr {N}),\dots ,\mathscr {C}_n(\mathscr {N}),\end{aligned}$$

we need a little bit more notation. Let $\mathscr {C}$ and $\mathscr {C}'$ be cluster systems on $X$. We write:

- $\mathscr {C} \vdash _{(1)} \mathscr {C}'$ if there exist two distinct clusters $A,B \in \mathscr {C}$ with $\mathscr {C}' = (\mathscr {C} - \{A,B\}) \cup \{A \cup B\}$ (see Fig. 3a).
- $\mathscr {C} \vdash _{(2)} \mathscr {C}'$ if there exist three pairwise distinct clusters $A,B,C \in \mathscr {C}$ with $\mathscr {C}' = (\mathscr {C} - \{A,B,C\}) \cup \{A \cup B,B \cup C\}$ (see Fig. 3b).

We will often use the simplified notation $\mathscr {C} \vdash \mathscr {C}'$ if either $\mathscr {C} \vdash _{(1)} \mathscr {C}'$ or $\mathscr {C} \vdash _{(2)} \mathscr {C}'$ holds in case it is not relevant which of the two conditions holds. A *maximal chain* on $X$ is a sequence $\mathscr {C}_1,\dots ,\mathscr {C}_n$ of $n$ cluster systems on $X$ such that

$$\begin{aligned} \{\{x\}: x \in X\} = \mathscr {C}_1 \vdash \mathscr {C}_2 \vdash \dots \vdash \mathscr {C}_n = \{X\}.\end{aligned}$$

The chains as defined above are maximal in the sense that the sequence contains *all* intermediate cluster systems that lead in a particular way from $\{\{x\} : x \in X\}$. In Section 5 we will consider more general chains where some intermediate cluster systems may be skipped. Before we state the main result of this section, we establish a useful property of cluster systems in a maximal chain on $X$.

### Lemma 3.1

Let $\mathscr {C}_1,\dots ,\mathscr {C}_n$ be a maximal chain on $X$ and $1 \le i \le n$. Then every cluster in $\mathscr {C}_i$ contains an element in *X* that is not contained in any other cluster in $\mathscr {C}_i$.

### Proof

We use induction on $i$. In the base case of the induction, $i=1$, we have $\mathscr {C}_1=\{\{x\} : x \in X\}$. Then, clearly, for all $x \in X$, the element $x$ is only contained in the cluster $\{x\}$.

Next consider the case $i>1$. By the definition of a maximal chain, we have $\mathscr {C}_{i-1} \vdash \mathscr {C}_i$. By induction, all clusters in $\mathscr {C}_{i-1}$ contain at least one element that is not contained in any other cluster in $\mathscr {C}_{i-1}$. But then, in view of the definition of $\vdash _{(1)}$ and $\vdash _{(2)}$, it follows that also all clusters in $\mathscr {C}_i$ contain at least one element that is not contained in any other cluster in $\mathscr {C}_i$, as required. $\square$

We now present our encoding for binary RTCNs.

### Theorem 3.2

Binary RTCNs on $X$ are in bijective correspondence with maximal chains on $X$.

### Proof

We have already seen that from every binary RTCN $\mathscr {N}$ on $X$ we obtain the maximal chain $\mathscr {C}_1(\mathscr {N}),\dots ,\mathscr {C}_n(\mathscr {N})$ on $X$.

So, assume that $\mathscr {C}_1,\dots ,\mathscr {C}_n$ is a maximal chain on $X$. To obtain a binary RTCN $\mathscr {N}$ on $X$ with $\mathscr {C}_i = \mathscr {C}_i(\mathscr {N})$ we use the maximal chain to guide the process of generating $\mathscr {N}$ during Steps $i=2,\dots ,n-1$:

- If $\mathscr {C}_{i-1} \vdash _{(1)} \mathscr {C}_i$ we perform (1).
- If $\mathscr {C}_{i-1} \vdash _{(2)} \mathscr {C}_i$ we perform (2).

It remains to show that the two vertices with in-degree 0 used when performing (1) and the three vertices with in-degree 0 used when performing (2), respectively, are uniquely determined by the maximal chain on $X$. But this follows immediately from the property of the cluster systems in a maximal chain on $X$ stated in Lemma 3.1, as this allows to uniquely determine the clusters involved in $\mathscr {C}_{i-1} \vdash \mathscr {C}_i$. $\square$

The encoding established in Theorem 3.2 is useful because it allows us to systematically break any binary RTCN on $X$ down into building blocks (i.e. cluster systems), which gives a simple way to understand the relationship between two binary RTCNs. We remark that there are two interesting special instances of our encoding:

- Maximal chains $\mathscr {C}_1,\dots ,\mathscr {C}_n$ on $X$ such that $\mathscr {C}_1 \vdash _{(1)} \mathscr {C}_2 \vdash _{(1)} \dots \vdash _{(1)} \mathscr {C}_n$ are in bijective correspondence with binary ranked trees on $X$.
- Let $\vdash ^*$ be the restricted variant of $\vdash$ defined by the additional requirements that:
  - For $\mathscr {C} \vdash ^*_{(1)} \mathscr {C}'$ to hold we must have
    - $$A \cap B \ne \emptyset$$, or
    - $A \cap C = \emptyset$ for all $C \in \mathscr {C} - \{A\}$, or
    - $$B \cap C = \emptyset$$ for all $$C \in \mathscr {C} - \{B\}$$.
  - For $\mathscr {C} \vdash ^*_{(2)} \mathscr {C}'$ to hold we must have
    - $A \cap D = \emptyset$ for all $D \in \mathscr {C} - \{A\}$, and
    - $B \cap D = \emptyset$ for all $D \in \mathscr {C} - \{B\}$, and
    - $$C \cap D = \emptyset$$ for all $$D \in \mathscr {C} - \{C\}$$.
  Then maximal chains $\mathscr {C}_1,\dots ,\mathscr {C}_n$ on $X$ such that $\mathscr {C}_1 \vdash ^* \mathscr {C}_2 \vdash ^* \dots \vdash ^* \mathscr {C}_n$ are in bijective correspondence with *binary ranked cactuses* on $X$, a proper subclass of binary RTCNs considered by Huber et al. (2024).

## Nearest neighbor interchange moves for binary RTCNs

In this section we explain how to use our encoding of binary RTCNs by maximal chains of cluster systems to compare unweighted binary RTCNs. One simple way to do this is to define the distance between two such networks $\mathscr {N}$ and $\mathscr {N}'$ to be

$$\begin{aligned} |\{\mathscr {C}_1(\mathscr {N}), \dots , \mathscr {C}_n(\mathscr {N})\} \triangle \{\mathscr {C}_1(\mathscr {N}'), \dots , \mathscr {C}_n(\mathscr {N}')\}| \end{aligned}$$

where $\triangle$ denotes the symmetric difference of sets. The metric on binary RTCNs arising in this way can be thought of as a ranked analogue of the Robinson-Foulds distance on rooted trees (Robinson and Foulds 1981).

**Fig. 4:**
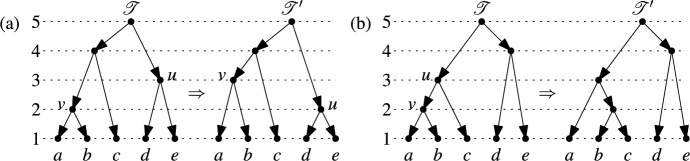
The two types of modifications on binary ranked trees allowed in an rNNI: (**a**) Swapping the ranks of vertices $u$ and $v$. (**b**) An actual nearest neighbor interchange

A more sophisticated approach is to define an analogue of the well-known nearest neighbor interchange distance for rooted phylogenetic trees (Robinson 1971). This distance has already been generalized to binary ranked trees by Gavryushkin et al. (2018) as follows. First, define two types of modifications of a binary ranked tree on $X$ (called *ranked nearest neighbor interchanges (rNNIs)*):

- For two vertices $u$ and $v$ with $\text {rank}(u) = \text {rank}(v) + 1$ and $(u,v)$ not an arc, the ranks of $u$ and $v$ are swapped without changing the topology of the tree (see Fig. 4a).
- For two vertices $u$ and $v$ with $\text {rank}(u) = \text {rank}(v) + 1$ and $(u,v)$ an arc, the topology of the tree is changed (see Fig. 4b).

Then, Gavryushkin et al. (2018) established the following result:

### Fact 4.1

For any two binary ranked trees $\mathscr {T}$ and $\mathscr {T}'$ on $X$ there exists a sequence of rNNIs that transform $\mathscr {T}$ into $\mathscr {T}'$.

Interestingly, as pointed out in the supplementary material by Collienne et al. (2021), there is a concise and uniform way to describe an rNNI between two binary ranked trees $\mathscr {T}$ and $\mathscr {T}'$ on $X$ using the corresponding maximal chains $\mathscr {C}_1,\dots ,\mathscr {C}_n$ and $\mathscr {C}'_1,\dots ,\mathscr {C}'_n$ on $X$: There exists $2 \le i \le n-1$ such that $\mathscr {C}_i \ne \mathscr {C}'_i$ and $\mathscr {C}_j = \mathscr {C}'_j$ for all $j \ne i$. Less formally, there is an rNNI between $\mathscr {T}$ and $\mathscr {T}'$ if the corresponding maximal chains differ in precisely one cluster system. For example, consider the two binary ranked trees $\mathscr {T}$ and $\mathscr {T}'$ on $X=\{a,b,c,d,e\}$ in Fig. 4a. Looking at the corresponding maximal chains on $X$ we have:

$$\begin{aligned} \mathscr {C}_1&= \{\{a\},\{b\},\{c\},\{d\},\{e\}\} = \mathscr {C}'_1\\ \mathscr {C}_2&= \{\{a,b\},\{c\},\{d\},\{e\}\} \ne \{\{a\},\{b\},\{c\},\{d,e\}\} = \mathscr {C}'_2\\ \mathscr {C}_3&= \{\{a,b\},\{c\},\{d,e\}\} = \mathscr {C}'_3\\ \mathscr {C}_4&= \{\{a,b,c\},\{d,e\}\} = \mathscr {C}'_4\\ \mathscr {C}_5&= \{\{a,b,c,d,e\}\} = \mathscr {C}'_5 \end{aligned}$$

**Fig. 5:**
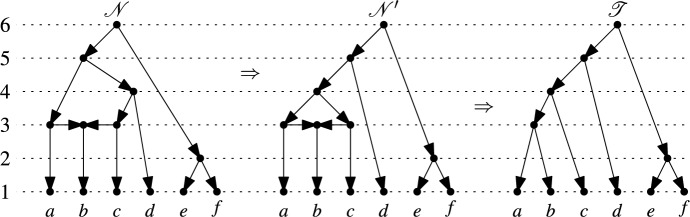
Two consecutive rNNI$^*$s that transform the binary RTCN $\mathscr {N}$ on $X=\{a,b,c,d,e,f\}$ through the intermediate binary RTCN $\mathscr {N}'$ to the binary ranked tree $\mathscr {T}$

While the description of rNNIs in terms of the binary ranked trees is very intuitive, it is not obvious how to directly generalize this to binary RTCNs. However, as with ranked trees, the description in terms of maximal chains on $X$ immediately suggests a way to do this: We say that there is a *ranked nearest neighbor interchange* between two binary RTCNs $\mathscr {N}$ and $\mathscr {N}'$ (both on $X$) if the corresponding maximal chains on $X$ differ in precisely one cluster system. We shall continue to use rNNI when referring to ranked nearest neighbor interchanges restricted to binary ranked trees as described above and will use rNNI$^*$ when referring to this generalization. Fig. 5 gives an example of what happens to the corresponding binary RTCNs when we apply such rNNI$^*$s; a complete list of all possible network changes that can occur is presented in Appendix 1.

We conclude this section by establishing that for any two binary RTCNs on $X$ there exists a sequence of rNNI$^*$s that transforms one into the other. This implies that we can define a distance between any pair of binary RTCNs by taking the length of a shortest sequence of rNNI$^*$s that transforms one of the networks to the other.

### Theorem 4.2

For any two binary RTCNs $\mathscr {N}_1$ and $\mathscr {N}_2$ on $X$ there exists a sequence of rNNI$^*$s that transform $\mathscr {N}_1$ into $\mathscr {N}_2$.

### Proof

Since every rNNI between two binary ranked trees on $X$ is also an rNNI$^*$ between them when we view the binary ranked trees as binary RTCNs, it suffices, by Fact 4.1, to show that for any binary RTCN $\mathscr {N}$ on $X$ there exists a sequence of rNNI$^*$s that transforms $\mathscr {N}$ into some binary ranked tree $\mathscr {T}$ on $X$ (Fig. 5 gives an example of such a sequence of rNNI$^*$s).

Let $\mathscr {C}_1, \dots , \mathscr {C}_n$ be the maximal chain on $X$ that corresponds to $\mathscr {N}$ by Theorem 3.2. The proof is by induction on the number $\ell$ of those $1 < j \le n$ with $\mathscr {C}_{j-1} \vdash _{(2)} \mathscr {C}_{j}$. In the base case of the induction, $\ell =0$, $\mathscr {N}$ is itself a binary ranked tree.

So, assume that $\ell > 0$. Let $i$ be the maximum of those $1 < j \le n$ with $\mathscr {C}_{j-1} \vdash _{(2)} \mathscr {C}_{j}$. By the definition of $\vdash _{(2)}$ there exist three pairwise distinct $A,B,C \in \mathscr {C}_{i-1}$ such that

$$\begin{aligned} \mathscr {C}_{i} = (\mathscr {C}_{i-1}-\{A,B,C\}) \cup \{A \cup B,B \cup C\}. \end{aligned}$$

By Lemma 3.1, we can select from each cluster in $\mathscr {C}_i$ an element that is unique to this cluster. Let $X' \subseteq X$ be the resulting subset of selected elements. To give an example, for the binary RTCN $\mathscr {N}$ in Fig. 5 we have $i=3$ and can select $X'=\{a,c,d,f\}$.

**Fig. 6:**
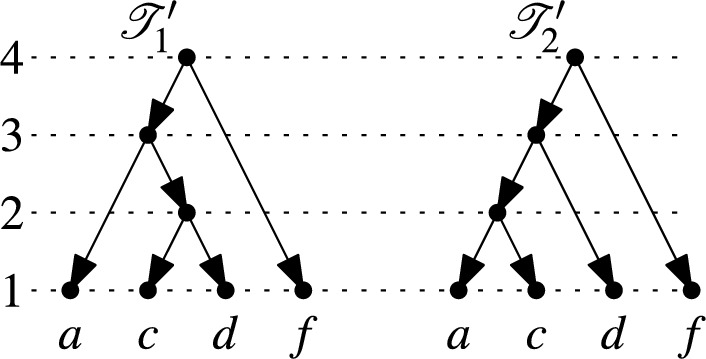
The ranked trees $\mathscr {T}_1'$ and $\mathscr {T}_2'$ referred to in the proof of Theorem 4.2

By the maximality of $i$, we have $\mathscr {C}_{j-1} \vdash _{(1)} \mathscr {C}_{j}$ for all $j > i$. Thus, after restricting all clusters to $X'$, the sequence $\mathscr {C}_{i},\dots ,\mathscr {C}_{n}$ becomes a maximal chain on $X'$ that only contains partitions of $X'$. This maximal chain on $X'$ corresponds, by Theorem 3.2, to a binary ranked tree $\mathscr {T}_1'$ on $X'$. In Fig. 6 the binary ranked tree $\mathscr {T}_1'$ resulting from the binary RTCN $\mathscr {N}$ in Fig. 5 is shown.

Let $y$ be the element in $X'$ selected from $A \cup B$ and let $z$ be the element in $X'$ selected from $B \cup C$. Let $\mathscr {T}_2'$ be a binary ranked tree on $X'$ that contains a vertex $u$ with $\text {rank}(u)=2$ and the arcs $(u,y)$ and $(u,z)$. Clearly, such a binary ranked tree exists and, by Fact 4.1, there exists a sequence of rNNIs that transforms $\mathscr {T}_1'$ into $\mathscr {T}_2'$. In Fig. 6, a suitable binary ranked tree $\mathscr {T}_2'$ is shown that arises by applying a single rNNI to $\mathscr {T}_1'$.

The sequence of rNNIs transforming $\mathscr {T}_1'$ into $\mathscr {T}_2'$ corresponds to a sequence of rNNI$^*$s that transform $\mathscr {N}$ into a binary RTCN $\mathscr {N}'$ such that all vertices of $\mathscr {N}$ with rank at most $i$ remain unchanged and only the vertices corresponding to the binary ranked tree $\mathscr {T}_1'$ are involved. In Fig. 5 the binary RTCN $\mathscr {N}'$ resulting from the rNNI between the binary ranked trees $\mathscr {T}_1'$ and $\mathscr {T}_2'$ in Fig. 6 is shown.

In preparation for the last step in the proof, we summarize the properties of the maximal chain on $X$ that corresponds to $\mathscr {N}'$:

- $$\mathscr {C}_j(\mathscr {N}') = \mathscr {C}_j(\mathscr {N})$$ for all $$1 \le j \le i$$
- $$\mathscr {C}_{j-1}(\mathscr {N}') \vdash _{(1)} \mathscr {C}_j(\mathscr {N}')$$ for all $$i < j \le n$$
- $$\mathscr {C}_{i+1}(\mathscr {N}') = (\mathscr {C}_{i}(\mathscr {N}') - \{A \cup B,B \cup C\}) \cup \{A \cup B \cup C\}$$

Now we perform the following rNNI$^*$ on $\mathscr {N}'$: We replace the cluster system $\mathscr {C}_i = \mathscr {C}_{i}(\mathscr {N}')$ by the cluster system

$$\begin{aligned} \mathscr {C}_i'' = (\mathscr {C}_{i-1}(\mathscr {N}') - \{A,B\}) \cup \{A \cup B\}. \end{aligned}$$

This is possible since $A,B,C \in \mathscr {C}_{i-1}(\mathscr {N}')$. Then we have

$$\begin{aligned} \mathscr {C}_{i+1}(\mathscr {N}') = (\mathscr {C}_i'' - \{A \cup B,C\}) \cup \{A \cup B \cup C\}.\end{aligned}$$

The resulting maximal chain on $X$ is

$$\begin{aligned} \mathscr {C}_1(\mathscr {N}') \vdash \dots \vdash \mathscr {C}_{i-1}(\mathscr {N}') \vdash _{(1)} \mathscr {C}_i'' \vdash _{(1)} \mathscr {C}_{i+1}(\mathscr {N}') \vdash _{(1)} \dots \vdash _{(1)} \mathscr {C}_{n}(\mathscr {N}').\end{aligned}$$

By Theorem 3.2, this maximal chain on $X$ corresponds to a binary RTCN $\mathscr {N}''$ on $X$. Moreover, by construction, the number of occurrences of $\vdash _{(2)}$ in this maximal chain on $X$ is $\ell -1$. Hence, by induction, there exists a sequence of rNNI$^*$s that transform $\mathscr {N}''$ into a binary ranked tree $\mathscr {T}$ on $X$. But then, there is also a sequence of rNNI$^*$s that transform $\mathscr {N}$ into $\mathscr {T}$. This finishes the inductive proof. In the example in Fig. 5 we have $\mathscr {N}'' = \mathscr {T}$. $\square$

### Corollary 4.3

For any two binary ranked trees $\mathscr {T}$ and $\mathscr {T}'$ on $X$, the length of a shortest sequence of rNNI$^*$s that transform $\mathscr {T}$ into $\mathscr {T}'$ is less than or equal to the length of a shortest sequence of rNNIs that transform $\mathscr {T}$ into $\mathscr {T}'$.

### Proof

Every rNNI between two binary ranked trees is an rNNI$^*$ between the two binary ranked trees (viewing the trees as binary RTCN). $\square$

## Non-binary ranked tree-child networks

So far we have shown how to compare binary RTCNs whose arcs are unweighted. In the introduction, in addition to binary RTCNs, we also informally introduced certain tree-child networks with non-negative arc weights, called ETCNs. In order to compare such ETCNs in the next section, we will need to consider non-binary rooted phylogenetic networks that are tree-child and have ranked vertices, as these can arise when shrinking down arcs to length zero. Similarly to other classes of non-binary networks (see e.g. Jetten and van Iersel (2016)), the formal definition of non-binary RTCNs involves taking a slightly more abstract view on the encoding for binary RTCNs. More specifically, in this section we define a certain partially ordered set or *poset* which not only allows us to say precisely what we mean by a non-binary RTCN, but to also define a distance on ETCNs in the next section.

Let $\mathfrak {T}(X)$ denote the set of all cluster systems on $X$ that occur in some maximal chain on $X$. For $\mathscr {C},\mathscr {C}' \in \mathfrak {T}(X)$ we write $\mathscr {C} \preceq \mathscr {C}'$ if there exists a maximal chain

$$\begin{aligned} \mathscr {C}_1 \vdash \mathscr {C}_2 \vdash \dots \vdash \mathscr {C}_n\end{aligned}$$

on $X$ with $\mathscr {C} = \mathscr {C}_i$ and $\mathscr {C}'= \mathscr {C}_j$ for some $1 \le i \le j \le n$. Then, by construction, $\preceq$ is a partial ordering on $\mathfrak {T}(X)$. We denote the resulting poset by $(\mathfrak {T}(X),\preceq )$. Note that an important question in this context is how to efficiently recognize and compare (with respect to $\preceq$) cluster systems in $\mathfrak {T}(X)$. But as this is not required in the remaining sections, we address this somewhat technical aspect of the poset in Appendix 2 for the interested reader.

A *chain* in $(\mathfrak {T}(X),\preceq )$ is a sequence $\mathscr {C}_1,\dots ,\mathscr {C}_t$ of $2 \le t \le n$ pairwise distinct cluster systems in $\mathfrak {T}(X)$ such that

$$\begin{aligned} \{\{x\}:x \in X\} = \mathscr {C}_1 \preceq \mathscr {C}_2 \preceq \dots \preceq \mathscr {C}_t = \{X\}.\end{aligned}$$

The integer $t$ is called the *length* of the chain. Thus, chains of length $n$ in $(\mathfrak {T}(X),\preceq )$ are precisely the maximal chains on $X$.

### Example 5.1

Consider $$X=\{a,b,\dots ,h\}$$. Then

$$\begin{aligned} \mathscr {C}_1&= \{\{a\},\{b\},\{c\},\{d\},\{e\},\{f\},\{g\},\{h\}\}\\ \mathscr {C}_2&= \{\{a,b,c,d\},\{c,d,e\},\{f\},\{g,h\}\}\\ \mathscr {C}_3&= \{\{a,b,c,d,e\},\{f,g,h\}\}\\ \mathscr {C}_4&= \{\{a,b,c,d,e,f,g,h\}\} \end{aligned}$$

is a chain of length 4 in $(\mathfrak {T}(X),\preceq )$.

In the proof of Theorem 3.2, we saw how a maximal chain on $X$ guides the process of generating the binary RTCN on $X$ that corresponds to the maximal chain. Here we generalize this idea to *all* chains in $(\mathfrak {T}(X),\preceq )$. Since we may no longer have $\mathscr {C}_i \vdash \mathscr {C}_{i+1}$ for two consecutive cluster systems in a chain, however, the process of generating the RTCN corresponding to a chain becomes a bit more complex to describe.

Let $\mathscr {C}_1,\dots ,\mathscr {C}_t$ be a chain in $(\mathfrak {T}(X),\preceq )$. The process of generating the corresponding RTCN consists of $t$ steps:

**Fig. 7:**
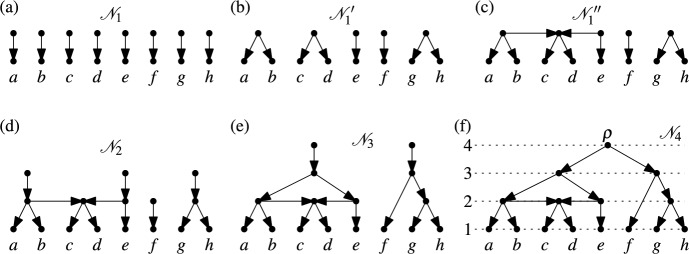
The process that generates a non-binary RTCN on $X=\{a,b,\dots ,h\}$ from the chain of length 4 in Example 5.1. (**a**) The result of performing Step 1. (**b**) The result of Phase 1 in Step 2. (**c**) The result of Phase 2 in Step 2. (**d**) The result of performing Step 2. (**e**) The result of performing Step 3. (**f**) The resulting non-binary RTCN after performing Step 4, the final step

- Step 1: For each $x \in X$ an arc with head $x$ is created. The tails of these arcs are pairwise distinct and form a set of $n$ vertices with in-degree 0 (see Fig. 7(a)).
- ${\underline{\text {Step }i (2 \le i \le t-1):}}$ Let $\mathscr {N}_{i-1}$ denote the network obtained at the end of Step $i-1$. For vertices $v$ of $\mathscr {N}_{i-1}$ we also use $C_v$ to denote the cluster on $X$ consisting of those $x \in X$ for which there exists a directed path in $\mathscr {N}_{i-1}$ from $v$ to $x$. There is a bijective correspondence between the vertices $v$ with in-degree 0 of $\mathscr {N}_{i-1}$ and the clusters in $\mathscr {C}_{i-1}$ obtained by mapping $v$ to $C_v$. For all $A \in \mathscr {C}_{i-1}$ put
  $$\begin{aligned}H(A) = \{B \in \mathscr {C}_i: A \subseteq B\}.\end{aligned}$$
  It follows from the definition of $\preceq$ that $H(A) \ne \emptyset$ for all $A \in \mathscr {C}_{i-1}$. Moreover, by Lemma 3.1, for all $B \in \mathscr {C}_i$ there exists some $A \in \mathscr {C}_{i-1}$ with $H(A) = \{B\}$. To illustrate the notation used to describe Step $i$, consider $i=2$ for Example 5.1 where we have:
  $$\begin{aligned} \quad \quad \quad H(\{a\})&= \{\{a,b,c,d\}\} = H(\{b\}), \ H(\{c\}) = \{\{a,b,c,d\},\{c,d,e\}\} = H(\{d\}),\\ \quad \quad \quad H(\{e\})&= \{\{c,d,e\}\}, \ H(\{f\}) = \{\{f\}\}, \ H(\{g\}) = \{\{g,h\}\} = H(\{h\}) \end{aligned}$$
  Step $i$ consists of three phases:
  - *Phase 1*: Any two vertices $v$ and $v'$ of $\mathscr {N}_{i-1}$ with in-degree 0 are identified if $H(C_v) = H(C_{v'})$. Let $\mathscr {N}_{i-1}'$ denote the resulting network (see Fig. 7(b)). For all vertices $u$ of $\mathscr {N}_{i-1}'$ with in-degree 0, let $H_u$ denote the set $H(C_v)$, where $v$ is any of the vertices of $\mathscr {N}_{i-1}$ with in-degree 0 that have been identified to form $u$.
  - *Phase 2*: For
    - all vertices $u$ of $\mathscr {N}_{i-1}'$ with in-degree 0 and $|H_u| \ge 2$, and
    - all vertices $u'$ of $\mathscr {N}_{i-1}'$ with in-degree 0, $|H_{u'}| = 1$ and $H_{u'} \subseteq H_u$
    add the arc with head $u$ and tail $u'$. Since $|H_u| \ge 2$, the vertices $u$ in this phase will become hybrid vertices. Let $\mathscr {N}_{i-1}''$ denote the resulting network (see Fig. 7c).
  - *Phase 3*: For all vertices $u$ of $\mathscr {N}_{i-1}''$ with in-degree 0 and out-degree at least 2, add a new arc with head $u$ and a new tail. This finishes Step $i$.
  At the end of Step $i$ we have a network $\mathscr {N}_i$ whose vertices with in-degree 0 are in bijective correspondence with the clusters in $\mathscr {C}_i$ (see Fig. 7d and e).
- ${\underline{\text {Step} \ n:}}$ All vertices with in-degree 0 in the network obtained after Step $t-1$ are identified as a single vertex which then forms the root $\rho$ of the resulting network (see Fig. 7f).

Finally, each vertex in the rooted phylogenetic network $\mathscr {N} = (V,E,\rho )$ on $X$ generated by the process described above is assigned a rank from the set $\{1,\dots ,t\}$ (see Fig. 7f) by putting:

- $$\text {rank}(x)=1$$ for all $$x \in X$$,
- $\text {rank}(u)=i$ for all vertices $u$ of the network $\mathscr {N}_{i}$ obtained at the end of Step $i$ such that $u$ is the head of an arc added in Step $i$ ($2 \le i \le t-1$),
- $$\text {rank}(\rho ) = t$$.

We now summarize the key properties of the rooted phylogenetic networks obtained by the process described above. The proof that these properties hold follows immediately from the construction of the network $\mathscr {N}$ from the given chain in $(\mathfrak {T}(X),\preceq )$.

### Theorem 5.2

For every chain $\mathscr {C}_1,\dots ,\mathscr {C}_t$ in $(\mathfrak {T}(X),\preceq )$ we obtain a rooted phylogenetic network $\mathscr {N}=(V,E,\rho )$ on $X$ together with a map $\textrm{rank} : V \rightarrow \{1,\dots ,t\}$ such that, for all $1 \le i < t$,

$$\begin{aligned} \mathscr {C}_i = \{C_v: \mathrm{there \ exists \ an \ arc} \ (u,v) \in E \ \textrm{with} \ \textrm{rank}(u) > i \ge \textrm{rank}(v)\},\end{aligned}$$

and $\mathscr {C}_t = \{C_{\rho }\} = \{X\}$. If $t=n$ (i.e. the chain is a maximal chain on $X$), $\mathscr {N}$ is the binary RTCN that corresponds to the chain by Theorem 3.2.

Note that, in the language of posets, $(\mathfrak {T}(X),\preceq )$ is *bounded* because we have

$$\begin{aligned} \{\{x\}:x \in X\} \preceq \mathscr {C} \preceq \{X\}\end{aligned}$$

for all $\mathscr {C} \in \mathfrak {T}(X)$. This, together with the fact that all maximal chains in $(\mathfrak {T}(X),\preceq )$ have the same length, implies that $(\mathfrak {T}(X),\preceq )$ is what is known as a *graded* poset (with the grading of cluster systems $\mathscr {C} \in \mathfrak {T}(X)$ given by $n-|\mathscr {C}|$). Moreover, Theorem 4.2 is equivalent to saying that $(\mathfrak {T}(X),\preceq )$ is *gallery-connected*. Note that a similar relationship for nearest neighbor interchanges on unrooted phylogenetic trees on $X$ appears in (Stadnyk 2022).

**Fig. 8:**
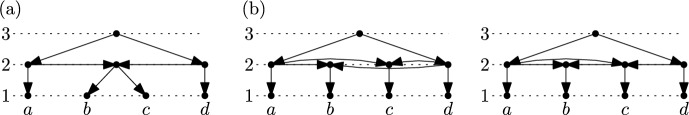
(**a**) The non-binary RTCN on $X=\{a,b,c,d\}$ that we obtain by Theorem 5.2 from the non-maximal chain $\mathscr {C}_1 = \{\{a\},\{b\},\{c\},\{d\}\}$, $\mathscr {C}_2 = \{\{a,b,c\},\{b,c,d\}\}$, $\mathscr {C}_3 = \{X\}$ in $(\mathfrak {T}(X),\preceq )$. (**b**) Two other non-binary tree-child networks with ranked vertices that also represent the structure of this chain

To conclude this section, we emphasize again that Theorem 5.2 only establishes that for each chain in the poset $(\mathfrak {T}(X),\preceq )$ the process described above produces a well-defined tree-child network with ranked vertices to represent this chain. In the following, we will refer to any network produced by this process as a (possibly non-binary) RTCN and, as a consequence, we have a one-to-one correspondence between chains in $(\mathfrak {T}(X),\preceq )$ and RTCNs. As can be seen in Fig. 8, however, for non-maximal chains the non-binary RTCN corresponding to that chain is usually only one among several different rooted phylogenetic networks that are tree-child, have ranked vertices and represent the structure of the chain. This highlights the fact that non-binary rooted phylogenetic networks that are tree-child and have ranked vertices are harder to capture than binary ones. In particular, a more complex encoding would need to be devised if one wanted to define a metric on *all* rooted phylogenetic networks that are tree-child and have ranked vertices. It could be interesting to explore this further in future work.

## Construction of a CAT(0)-orthant space of ETCNs

In this section, we define a distance on the collection of binary ETCNs having the same leaf set. The main idea is to use the poset $(\mathfrak {T}(X),\preceq )$ introduced in Section 5 to define a continuous space of such networks and, by using properties of $(\mathfrak {T}(X),\preceq )$, show that this space is a so called CAT(0)-orthant space.

**Fig. 9:**
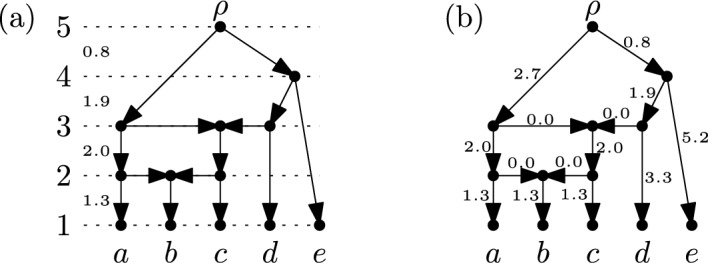
(**a**) A binary RTCN on $X=\{a,b,c,d,e\}$ where positive real-valued differences between consecutive ranks are given. (**b**) The corresponding equidistant weighting of the arcs of the network

First, we need to present some more definitions. We call a non-negative weighting of the arcs in a binary RTCN $\mathscr {N}$ on $X$ *equidistant* if the total weight of the arcs in a directed path in $\mathscr {N}$ from $\rho$ to some $x \in X$ does not depend on the choice of $x$ and the directed path (see e.g. Fig. 9). Note that, given non-negative real-valued differences between consecutive ranks, an equidistant weighting is obtained by assigning to each arc the total difference between the rank of its head and tail. Conversely, every equidistant weighting of the arcs that is *consistent* with the ranks of its vertices (i.e. vertices of the same rank have the same distance from the root and the higher the rank of a vertex the smaller the distance of it from the root), clearly yields corresponding non-negative, real-valued differences between consecutive ranks. Thus, to describe all equidistant weightings of a binary RTCN that are consistent with the ranks of its vertices, it suffices to look at all possible ways to assign non-negative real-valued differences between consecutive ranks.

To make this more precise, we use again the fact that, by Theorem 3.2, binary RTCNs on $X$ are in bijective correspondence with maximal chains

$$\begin{aligned}\{\{x\}:x \in X\}=\mathscr {C}_1,\dots ,\mathscr {C}_n=\{X\}\end{aligned}$$

on $X$. Assigning positive, real-valued differences between consecutive ranks then corresponds to a map $\omega$ that assigns, for all $1 \le i < n$, to the cluster system $\mathscr {C}_i$ a positive real number $\omega (\mathscr {C}_i)$. To illustrate this, consider again the example in Fig. 9a, where we obtain the following map $\omega$:

$$\begin{aligned} \omega (\mathscr {C}_1)&= \omega (\{\{a\},\{b\},\{c\},\{d\},\{e\}\}) = 1.3,\\ \omega (\mathscr {C}_2)&= \omega (\{\{a,b\},\{b,c\},\{d\},\{e\}\}) = 2.0,\\ \omega (\mathscr {C}_3)&= \omega (\{\{a,b,c\},\{b,c,d\},\{e\}\}) = 1.9,\\ \omega (\mathscr {C}_4)&= \omega (\{\{a,b,c\},\{b,c,d,e\}\}) = 0.8. \end{aligned}$$

**Fig. 10:**
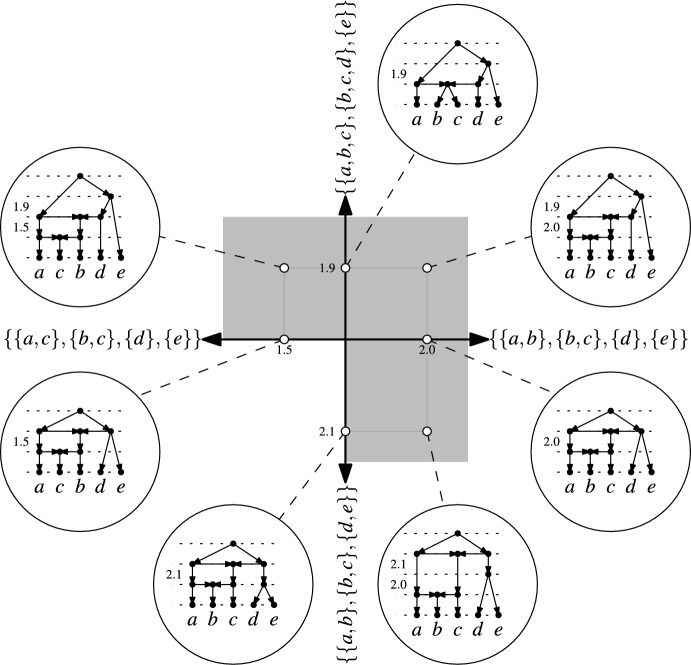
The gray squares represent three orthants of maximum dimension in the orthant space $\mathfrak {S}(X)$ for $X=\{a,b,c,d,e\}$. These orthants are actually 4-dimensional. They are projected into the plane by showing only two of the four coordinate axes that determine each of them (each coordinate axis is labeled by a cluster system in $\mathfrak {T}(X)$; the two axes corresponding to the cluster systems $\{\{a\},\{b\},\{c\},\{d\},\{e\}\}$ and $\{\{a,b,c\},\{b,c,d,e\}\}$ are not shown in the projection). Each point in an orthant corresponds to an ETCN on $X$ with the coordinates of the point describing the difference between consecutive ranks

The maps $\omega$ for a fixed binary RTCN form an $(n-1)$-dimensional *orthant* in $\mathbb {R}^{(n-1)}$ that is spanned by the $n-1$ axes that each correspond to one of the cluster systems $\mathscr {C}_1,\dots ,\mathscr {C}_{n-1}$. For example, the orthant for the binary RTCN in Fig. 9a is illustrated in Fig. 10 along with the orthants for two other binary RTCNs. Orthants for different binary RTCNs may share some of their axes. This is the case precisely when the corresponding maximal chains on $X$ share some of its cluster systems. Intuitively, as can be seen in Fig. 10, orthants are “glued” together along these shared axes and, in this way, we obtain a continuous space whose points are meant to represent binary RTCNs on $X$ with an equidistant weighting of its arcs that is consistent with the ranks of the vertices.

One technical aspect, however, also illustrated in Fig. 10, is that points that lie on the boundary of an orthant correspond to maps $\omega$ that assign $0$ to certain cluster systems. Intuitively, this means that the cluster system is skipped, leading to a (non-maximal) chain in the poset $(\mathfrak {T}(X),\preceq )$ which then corresponds to a (not necessarily binary) RTCN on $X$ obtained by Theorem 5.2. In view of this, we call a (not necessarily binary) RTCN $\mathscr {N}$ obtained by Theorem 5.2 together with an equidistant weighting of the arcs of $\mathscr {N}$ that is consistent with the ranks of the vertices of $\mathscr {N}$ an *equidistant tree-child network* (ETCN) on $X$.

A concise, formal description of the continuous space we have just described can be obtained by considering maps $\omega : \mathfrak {T}(X)-\{\{X\}\} \rightarrow \mathbb {R}_{\ge 0}$. For such a map, put $\textrm{supp}(\omega ) = \{\mathscr {C} \in \mathfrak {T}(X) : \omega (\mathscr {C}) > 0\}.$ Then the *orthant-space* $\mathfrak {S}(X)$ of all ETCNs on $X$ consists of all maps $\omega : \mathfrak {T}(X)-\{\{X\}\} \rightarrow \mathbb {R}_{\ge 0}$ such that the cluster systems in $\textrm{supp}(\omega )$ (together with the cluster systems $\{\{x\}:x \in X\}$ and $\{X\}$), when ordered by $\preceq$, form a chain in the poset $(\mathfrak {T}(X),\preceq )$. More details about the general construction of an orthant-space based on the chains in a poset can be found in (Huber et al. (2024), Sec. 4.1). We remark that this construction can also be used to obtain the space of ultrametric trees presented by Gavryushkin and Drummond (2016) (cf. Huber et al. (2024)).

We now show that the space $\mathfrak {S}(X)$ comes equipped with a distance that has some attractive properties. More specifically, in the theorem below we show that $\mathfrak {S}(X)$ together with the distance $\delta$ that assigns the length $\delta (\omega ,\omega ')$ of a shortest path^1^ or *geodesic* between any two points $\omega , \omega '$ in $\mathfrak {S}(X)$ is a *CAT(0)-orthant space*. Note that this immediately implies that there is a unique geodesic between any two points in $\mathfrak {S}(X)$. As it is quite technical and not important for the proof, we shall not present the definition of CAT(0)-orthant spaces here, but instead refer the reader to e.g. (Miller et al. (2015), Section 6) for more details.

### Theorem 6.1

The metric space $(\mathfrak {S}(X),\delta )$ is a CAT(0)-orthant space whose points are in bijective correspondence with ETCNs on $X$.

### Proof

It is known^2^ (see e.g. (Huber et al. (2024), Sec. 4.1) for more details), that constructing a metric space based on a poset in the way that $(\mathfrak {S}(X),\delta )$ was constructed based on the poset $(\mathfrak {T}(X),\preceq )$ always yields a CAT(0)-orthant space.

We now show that the points in $(\mathfrak {S}(X),\delta )$ are in bijective correspondence with ETCNs on $X$. First note that each point $\omega \in \mathfrak {S}(X)$ corresponds to a chain $\mathscr {C}_1, \dots , \mathscr {C}_t$ in $(\mathfrak {T}(X),\preceq )$ that is obtained by ordering the cluster systems in $\textrm{supp}(\omega )$ together with the cluster systems $\{\{x\}:x \in X\}$ and $\{X\}$ by $\preceq$. By Theorem 5.2, the chain yields a well-defined (but not necessarily binary) RTCN on $X$. From the values $\omega (\mathscr {C}_i)$, $1 \le i < t$, we obtain an equidistant weighting of the arcs of this RTCN that is consistent with the ranks of the vertices as described in this section.

Conversely, assume we are given an ETCN on $X$, that is, a (not necessarily binary) RTCN $\mathscr {N}$ on $X$ together with an equidistant weighting of the arcs that is consistent with the ranks of the vertices of $\mathscr {N}$. Let $\mathscr {C}_1, \dots , \mathscr {C}_t$ be the chain in $(\mathfrak {T}(X),\preceq )$ that corresponds to $\mathscr {N}$ by Theorem 5.2. As described in the text, the given equidistant weighting of the arcs of $\mathscr {N}$ yields non-negative values $\omega (\mathscr {C}_i)$ for all $1 \le i < t$. We formally extend these to a map $\omega : \mathfrak {T}(X) - \{\{X\}\} \rightarrow \mathbb {R}_{\ge 0}$ by putting $\omega (\mathscr {C})=0$ for all $\mathscr {C} \in \mathfrak {T}(X) - \{\mathscr {C}_1, \dots , \mathscr {C}_{t-1},\{X\}\}$, which then yields the point in $\mathfrak {S}(X)$ corresponding to the given ETCN. $\square$

It follows immediately from Theorem 6.1, that the distance between any two ETCNs on $X$, that is, the value $\delta (\omega ,\omega ')$ for the corresponding maps $\omega ,\omega ' \in \mathfrak {S}(X)$, can be computed in polynomial time (Miller et al. (2015), Corollary 6.19). In addition, we have the following corollary about distances in the space $\mathfrak {S}(X)$.

### Corollary 6.2

Let $\omega _1,\omega _2 \in \mathfrak {S}(X)$ be points that correspond to ultrametric trees $\mathscr {T}_1$ and $\mathscr {T}_2$, respectively. Then $\delta (\omega _1,\omega _2)$ equals the distance between $\mathscr {T}_1$ and $\mathscr {T}_2$ in the space of ultrametric trees by Gavryushkin and Drummond (2016).

### Proof

Let $\omega$ be a point on the unique geodesic in $\mathfrak {S}(X)$ between $\omega _1$ and $\omega _2$. Since $\mathfrak {S}(X)$ is a CAT(0)-orthant space, it follows from (Miller et al. (2015), Corollary 6.19) that $\textrm{supp}(w) \subseteq \textrm{supp}(\omega _1) \cup \textrm{supp}(\omega _2)$. Hence, $\omega$ corresponds to an ultrametric tree. Thus, each point on the unique geodesic in $\mathfrak {S}(X)$ between $\omega _1$ and $\omega _2$ corresponds to an ultrametric tree, implying that this geodesic corresponds to the unique geodesic in the space of ultrametric trees by Gavryushkin and Drummond (2016) between $\mathscr {T}_1$ and $\mathscr {T}_2$. In particular, the corresponding geodesics in the two spaces have the same length, as required. $\square$

## Conclusion

In this paper, we have presented various ways to compare binary RTCNs. Interestingly, it is shown by Collienne and Gavryushkin (2021) that, given two binary ranked trees $\mathscr {T}_1$ and $\mathscr {T}_2$ on $X$, the rNNI-distance between $\mathscr {T}_1$ and $\mathscr {T}_2$ can be computed in polynomial time. It would be nice to know if the analogous rNNI$^*$-distance between two binary RTCNs defined in Sect. 4 can also be computed in polynomial time. In addition to this, it remains open if Corollary 4.3 can be strengthened to equality always holding for the lengths of the two sequences.

In another direction, it would be of interest to investigate if alternative distances on RTCNs can be defined by generalizing other types of ranked tree modifications (for example, *subtree prune and regraft operations (SPRs)* considered by Collienne et al. (2024)), and to also see if the ranked tree distances considered by Kim et al. (2020) might be generalized to RTCNs. Instead of looking at other distances on RTCNs, it could also be worth investigating if alternative continuous network spaces can be defined for different classes of networks (e.g. networks where the vertices have a different type of ranking such as HGT-consistent labelings (van Iersel et al. 2022)).

Another avenue of research is to further consider combinatorial and topological properties of the poset $(\mathfrak {T}(X),\preceq )$. For example, we have shown that this poset is gallery-connected, a property that, for *any* finite poset, is immediately implied in case the poset is *shellable* (see e.g. Björner and Wachs (1983) for a formal definition of shellability). Is $(\mathfrak {T}(X),\preceq )$ shellable? If this were true, then it would immediately imply that the space $(\mathfrak {S}(X),\delta )$ considered in Sect. 6 has some special topological properties. Note that a similar combinatorial technique was used by Ardila and Klivans (2006) to understand the topology of spaces of (unranked) equidistant trees.

Finally, Theorem 6.1 implies that methods for performing a variety of statistical computations (e.g. Fréchet mean and variance (Bacák 2014; Miller et al. 2015), an analogue of partial principal component analysis (Nye et al. 2017) and confidence sets (Willis 2019)) can be applied (or extended) to the metric space $(\mathfrak {S}(X),\delta )$. These methods allow, for example, the computation of a *consensus* for a collection of ETCNs. It would be interesting to further explore this possibility, and also to investigate geometric properties of the space $(\mathfrak {S}(X),\delta )$.

## Figures and Tables

**Fig. 11:**
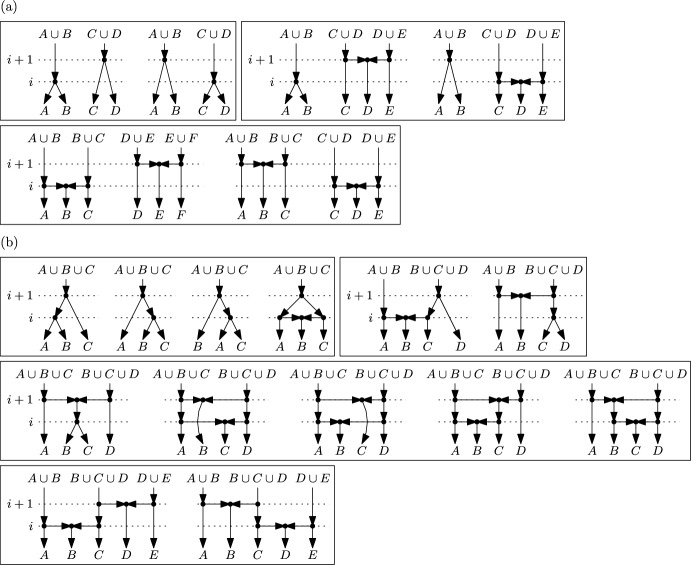
Changes in a binary RTCN that are the result of a single ranked nearest neighbor interchange as defined in the main text. Each box depicts all configurations that can be obtained in a single move starting from any one of the configurations in that box. (**a**) Moves that only affect the ranks of vertices but not the topology of the network. (**b**) Moves that also affect the topology of the network

## Data Availability

Data sharing not applicable to this article as no datasets were generated or analyzed during the current study.

## Appendix 1

In this appendix we present a complete list of the possible types of network modifications that can occur when performing a ranked nearest neighbor interchange on a binary RTCN (see Fig. 11). This overview was obtained by systematically considering all possible configurations that can occur in cluster systems $\mathscr {C},\mathscr {C}',\mathscr {C}'' \in \mathfrak {T}(X)$ with $\mathscr {C} \vdash \mathscr {C}' \vdash \mathscr {C}''$. Note that there are at most six clusters in $\mathscr {C} - \mathscr {C}''$. We focus on these clusters and on which unions of them may be formed when performing $\mathscr {C} \vdash \mathscr {C}'$ and then $\mathscr {C}' \vdash \mathscr {C}''$. This is then displayed in Fig. 11 as a corresponding network structure in a binary RTCN. In this figure, $A,B,C, \dots$ denote the clusters in $\mathscr {C}-\mathscr {C}''$.

## Appendix 2

In this appendix we present three technical lemmas concerning the poset $\mathfrak {T}(X)$ and briefly explain how they can be used to decide efficiently (i.e. in polynomial time with respect to $|X|$)

1. if a given cluster system $\mathscr {C}'$ on $X$ is contained in $\mathfrak {T}(X)$, and
2. if two given cluster systems $\mathscr {C}, \mathscr {C}' \in \mathfrak {T}(X)$ satisfy $\mathscr {C} \preceq \mathscr {C}'$.

The first lemma describes the relevant configurations in cluster systems. We write $\mathscr {C} \prec \mathscr {C}'$ for two cluster systems $\mathscr {C}, \mathscr {C}' \in \mathfrak {T}(X)$ if $\mathscr {C} \preceq \mathscr {C}'$ and $\mathscr {C} \ne \mathscr {C}'$.

### Lemma 7.1

Let $\mathscr {C}, \mathscr {C}' \in \mathfrak {T}(X)$ with $\mathscr {C} \prec \mathscr {C}'$. Then one of the following must hold:

(i) There exist two distinct $a,b \in X$ such that there exist distinct unique clusters $A,B \in \mathscr {C}$ with $a \in A$, $b \in B$ and, for all $C' \in \mathscr {C}'$, $\{a,b\} \cap C' \in \{\emptyset ,\{a,b\}\}$.
(ii) There exist three pairwise distinct $a,b,c \in X$ such that there exist pairwise distinct unique clusters $A,B,C \in \mathscr {C}$ with $a \in A$, $b \in B$, $c \in C$ and, for all $C' \in \mathscr {C}'$, $\{a,b,c\} \cap C' \in \{\emptyset ,\{a,b\},\{b,c\},\{a,b,c\}\}$.

### Proof

By Lemma 3.1, every cluster in $\mathscr {C}$ contains an element unique to this cluster. Since $\mathscr {C} \prec \mathscr {C}'$, there must exist $\mathscr {C}'' \in \mathfrak {T}(X)$ with $\mathscr {C} \vdash \mathscr {C}'' \preceq \mathscr {C}'$. Thus, we have either $\mathscr {C} \vdash _{(1)} \mathscr {C}''$, implying (i), or $\mathscr {C} \vdash _{(2)} \mathscr {C}''$, implying (ii). $\square$

The next lemma establishes that the configuration described in Lemma 7.1(i), if present, can always be used to start a chain.

### Lemma 7.2

Let $\mathscr {C}, \mathscr {C}' \in \mathfrak {T}(X)$ with $\mathscr {C} \prec \mathscr {C}'$ and let $a,b \in X$ be two distinct elements such that there exist distinct unique clusters $A,B \in \mathscr {C}$ with $a \in A$, $b \in B$ and, for all $C' \in \mathscr {C}'$, $\{a,b\} \cap C' \in \{\emptyset ,\{a,b\}\}$. Then $(\mathscr {C} - \{A,B\}) \cup \{A \cup B\} \preceq \mathscr {C}'$.

### Proof

Since $\mathscr {C} \prec \mathscr {C}'$, there exists a chain

$$\begin{aligned}\mathscr {C} = \mathscr {C}_0 \vdash \mathscr {C}_1 \vdash \dots \vdash \mathscr {C}_k = \mathscr {C}'\end{aligned}$$

with $k \ge 1$. We use induction on $k$. In the base case of the induction, $k=1$, we have $\mathscr {C}' = (\mathscr {C} - \{A,B\}) \cup \{A \cup B\}$, as required.

Now assume that $k \ge 2$. We perform a case analysis on $\mathscr {C}_1$.

- $\mathscr {C}_1 = (\mathscr {C}_0 - \{A,B\}) \cup \{A \cup B\}$: Then we are done.
- $\mathscr {C}_1 = (\mathscr {C}_0 - \{E,F\}) \cup \{E \cup F\}$ for two distinct $E,F \in \mathscr {C}_0 - \{A,B\}$: Put $\mathscr {C}'_2 = (\mathscr {C}_1 - \{A,B\}) \cup \{A \cup B\}$. By induction, $\mathscr {C}'_2 \preceq \mathscr {C}'$. Thus, putting $\mathscr {C}'_1 = (\mathscr {C}_0 - \{A,B\}) \cup \{A \cup B\}$, we have
  $$\begin{aligned}\mathscr {C}_0 \vdash \mathscr {C}'_1 \vdash (\mathscr {C}'_1 - \{E,F\}) \cup \{E \cup F\} = \mathscr {C}'_2 \preceq \mathscr {C}',\end{aligned}$$
  as required.
- $\mathscr {C}_1 = (\mathscr {C}_0 - \{B,E\}) \cup \{B \cup E\}$ for some $E \in \mathscr {C}_0 - \{A,B\}$: Put $\mathscr {C}'_2 = (\mathscr {C}_1 - \{A,B \cup E\}) \cup \{A \cup B \cup E\}$. By induction, $\mathscr {C}'_2 \preceq \mathscr {C}'$. Thus, putting $\mathscr {C}'_1 = (\mathscr {C}_0 - \{A,B\}) \cup \{A \cup B\}$, we have
  $$\begin{aligned}\mathscr {C}_0 \vdash \mathscr {C}'_1 \vdash (\mathscr {C}'_1 - \{A \cup B,E\}) \cup \{A \cup B \cup E\} = \mathscr {C}'_2 \preceq \mathscr {C}',\end{aligned}$$
  as required.
- $\mathscr {C}_1 = (\mathscr {C}_0 - \{E,F,G\}) \cup \{E \cup F,F \cup G\}$ for three pairwise distinct $E,F,G \in \mathscr {C}_0 - \{A,B\}$: Put $\mathscr {C}'_2 = (\mathscr {C}_1 - \{A,B\}) \cup \{A \cup B\}$. By induction, $\mathscr {C}'_2 \preceq \mathscr {C}'$. Thus, putting $\mathscr {C}'_1 = (\mathscr {C}_0 - \{A,B\}) \cup \{A \cup B\}$, we have
  $$\begin{aligned}\mathscr {C}_0 \vdash \mathscr {C}'_1 \vdash (\mathscr {C}'_1 - \{E,F,G\}) \cup \{E \cup F,F \cup G\} = \mathscr {C}'_2 \preceq \mathscr {C}',\end{aligned}$$
  as required.
- $\mathscr {C}_1 = (\mathscr {C}_0 - \{B,E,F\}) \cup \{B \cup E,E \cup F\}$ for two distinct $E,F \in \mathscr {C}_0 - \{A,B\}$: Put $\mathscr {C}'_2 = (\mathscr {C}_1 - \{A,B \cup E\}) \cup \{A \cup B \cup E\}$. By induction, $\mathscr {C}'_2 \preceq \mathscr {C}'$. Thus, putting $\mathscr {C}'_1 = (\mathscr {C}_0 - \{A,B\}) \cup \{A \cup B\}$, we have
  $$\begin{aligned}\mathscr {C}_0 \vdash \mathscr {C}'_1 \vdash (\mathscr {C}'_1 - \{A \cup B,E,F\}) \cup \{A \cup B \cup E,E \cup F\} = \mathscr {C}'_2 \preceq \mathscr {C}',\end{aligned}$$
  as required.
- $\mathscr {C}_1 = (\mathscr {C}_0 - \{B,E,F\}) \cup \{E \cup B,B \cup F\}$ for two distinct $E,F \in \mathscr {C}_0 - \{A,B\}$ or $\mathscr {C}_1 = (\mathscr {C}_0 - \{A,B,E\}) \cup \{A \cup B,B \cup E\}$ for some $E \in \mathscr {C}_0 - \{A,B\}$: For $2 \le i \le k$, put
  $$\begin{aligned} \mathscr {C}'_i = \{\cup _{C \in \mathscr {C}-\{B\},C \subseteq D} \, C: D \in \mathscr {C}_i\} \cup \{B\}.\end{aligned}$$
  Then, intuitively, $\mathscr {C}_0 \vdash \mathscr {C}'_2 \vdash \dots \vdash \mathscr {C}'_k$ is the chain obtained by skipping the step $\mathscr {C}_0 \vdash \mathscr {C}_1$. Moreover, since $\{a,b\} \cap C' \in \{\emptyset ,\{a,b\}\}$ for all $C' \in \mathscr {C}'$, we have
  $$\begin{aligned}\mathscr {C}' = \{D: D \in \mathscr {C}'_k - \{B\}, \ a \not \in D\} \cup \{D \cup B: D \in \mathscr {C}'_k, \ a \in D\}\end{aligned}$$
  Hence, putting $\mathscr {C}''_1 = (\mathscr {C} - \{A,B\}) \cup \{A \cup B\}$ and, for $2 \le i \le k$,
  $$\begin{aligned}\mathscr {C}''_i = \{D: D \in \mathscr {C}'_i - \{B\}, \ a \not \in D\} \cup \{D \cup B: D \in \mathscr {C}'_i, \ a \in D\},\end{aligned}$$
  we obtain the chain $\mathscr {C}_0 \vdash \mathscr {C}''_1 \vdash \mathscr {C}''_2 \vdash \dots \vdash \mathscr {C}''_k = \mathscr {C}'$, as required.
- $\mathscr {C}_1 = (\mathscr {C}_0 - \{A,B,E\}) \cup \{A \cup E,E \cup B\}$ for some $E \in \mathscr {C}_0 - \{A,B\}$: Put $\mathscr {C}'_2 = (\mathscr {C}_1 - \{A \cup E,B \cup E\}) \cup \{A \cup B \cup E\}$. By induction, $\mathscr {C}'_2 \preceq \mathscr {C}'$. Thus, putting $\mathscr {C}'_1 = (\mathscr {C}_0 - \{A,B\}) \cup \{A \cup B\}$, we have
  $$\begin{aligned}\mathscr {C}_0 \vdash \mathscr {C}'_1 \vdash (\mathscr {C}'_1 - \{A \cup B,E\}) \cup \{A \cup B \cup E\} = \mathscr {C}'_2 \preceq \mathscr {C}',\end{aligned}$$
  as required.

$$\square$$

The next lemma establishes that if the configuration described in Lemma 7.1(i) is not present, we can always select a suitable instance of the configuration described in Lemma 7.1(ii) to start a chain.

### Lemma 7.3

Let $\mathscr {C}, \mathscr {C}' \in \mathfrak {T}(X)$ with $\mathscr {C} \prec \mathscr {C}'$ and there are no two distinct elements $a',b' \in X$ such that there exist distinct unique clusters $A',B' \in \mathscr {C}$ with $a' \in A'$, $b' \in B'$ and, for all $C' \in \mathscr {C}'$, $\{a',b'\} \cap C' \in \{\emptyset ,\{a',b'\}\}$. Moreover, let $a,b,c \in X$ be three pairwise distinct elements such that there exist pairwise distinct unique clusters $A,B,C \in \mathscr {C}$ with $a \in A$, $b \in B$, $c \in C$ and, for all $C' \in \mathscr {C}'$, $\{a,b,c\} \cap C' \in \{\emptyset ,\{a,b\},\{b,c\},\{a,b,c\}\}$. Let $a,b,c$ be chosen such that $\{C' \in \mathscr {C}' : b \in C'\}$ is maximal with respect to set inclusion. Then $(\mathscr {C} - \{A,B,C\}) \cup \{A \cup B,B \cup C\} \preceq \mathscr {C}'$.

### Proof

Since $\mathscr {C} \prec \mathscr {C}'$, there exists a chain

$$\begin{aligned} \mathscr {C} = \mathscr {C}_0 \vdash \mathscr {C}_1 \vdash \dots \vdash \mathscr {C}_k = \mathscr {C}' \end{aligned}$$

with $$k \ge 1$$.

Let $\ell$ be maximum such that $B \in \mathscr {C}_\ell$. By the assumptions in the statement of the lemma, we have $0 \le \ell < k$. Moreover, since there are no two distinct elements $a',b' \in X$ such that there exist distinct unique clusters $A',B' \in \mathscr {C}_0$ with $a' \in A'$, $b' \in B'$ and, for all $C' \in \mathscr {C}'$, $\{a',b'\} \cap C' \in \{\emptyset ,\{a',b'\}\}$, we must have $\mathscr {C}_{\ell } \vdash _{(2)} \mathscr {C}_{\ell +1}$. We consider two cases.

$\mathscr {C}_{\ell +1} = (\mathscr {C}_\ell - \{B,E,F\}) \cup \{B \cup E, E \cup F\}$ for two distinct $E,F \in \mathscr {C}_\ell - \{B\}$: Let $e \in E$ and $f \in F$ be elements unique to these clusters in $\mathscr {C}_\ell$, which must exist by Lemma 3.1. This implies that there exist distinct unique $E',F' \in \mathscr {C}_0$ with $e \in E'$ and $f \in F'$ and, for all $C' \in \mathscr {C}_k$, $\{b,e,f\} \cap C' \in \{\emptyset ,\{b,e\},\{e,f\},\{b,e,f\}\}$. Hence, $\{C' \in \mathscr {C}_k : b \in C'\} \subseteq \{C' \in \mathscr {C}_k : e \in C'\}$. Thus, by the maximality of the choice of $a,b,c$, the two distinct elements $c,e \in X$ are such that, for all $C' \in \mathscr {C}_k$, we have $\{b,e\} \cap C' \in \{\emptyset ,\{b,e\}\}$, a contradiction. Hence, this case cannot occur.

$\mathscr {C}_{\ell +1} = (\mathscr {C}_\ell - \{B,E,F\}) \cup \{E \cup B,B \cup F\}$ for two distinct $E,F \in \mathscr {C}_\ell - \{B\}$: We consider the chain $\mathscr {C}'_1 \vdash \mathscr {C}'_2 \vdash \dots \vdash \mathscr {C}'_{k}$ obtained by, intuitively, skipping the step $\mathscr {C}_{\ell } \vdash \mathscr {C}_{\ell +1}$ in the chain $\mathscr {C}_0 \vdash \mathscr {C}_1 \vdash \dots \vdash \mathscr {C}_k$, which causes $B$ to remain a separate cluster. More precisely, we put

$$\begin{aligned} \mathscr {C}'_i = {\left\{ \begin{array}{ll} \mathscr {C}_{i-1} & \text {if} \ 1 \le i \le \ell +1\\ \{\cup _{G \in \mathscr {C} - \{B\}, G \subseteq D} \, G: D \in \mathscr {C}_{i}\} \cup \{B\} & \text {if} \ \ell +2 \le i \le k. \end{array}\right. } \end{aligned}$$

Thus, by the choice of $a,b,c$, we have

$$\begin{aligned}\mathscr {C}_k = \{D: D \in \mathscr {C}'_k - \{B\}, \ \{a,c\} \cap D = \emptyset \} \cup \{D \cup B: D \in \mathscr {C}'_k, \ \{a,c\} \cap D \ne \emptyset \}.\end{aligned}$$

But then we also have the chain

$$\begin{aligned}\mathscr {C}''_0 \vdash \mathscr {C}''_1 \vdash \dots \vdash \mathscr {C}''_k\end{aligned}$$

obtained by putting

$$\begin{aligned} \mathscr {C}''_i = {\left\{ \begin{array}{ll} \mathscr {C} & \text {if} \ i=0\\ (\mathscr {C} - \{A,B,C\}) \cup \{A \cup B,B \cup C\} & \text {if} \ i=1\\ \{D: D \in \mathscr {C}'_i-\{B\}, \ \{a,c\} \cap D = \emptyset \} \cup \{D \cup B: D \in \mathscr {C}'_i, \ \{a,c\} \cap D \ne \emptyset \} & \text {if} \ 2 \le i \le k, \end{array}\right. } \end{aligned}$$

as required. $$\square$$

We now sketch how these three lemmas can be used to decide (P1) and (P2) in polynomial time. Noting that $\{\{x\} : x \in X\} \preceq \mathscr {C}'$ must hold for all cluster systems $\mathscr {C}' \in \mathfrak {T}(X)$, Problem (P1) can be solved by putting $\mathscr {C} = \{\{x\} : x \in X\}$, tentatively assuming that $\mathscr {C}' \in \mathfrak {T}(X)$ and applying Lemmas 7.2 and 7.3 recursively until either we arrive at the cluster system $\mathscr {C}'$, implying that $\mathscr {C}' \in \mathfrak {T}(X)$, or we arrive at a cluster system $\mathscr {C}''$ where we get stuck (cannot apply Lemmas 7.2 and 7.3 any more or $|\mathscr {C}''| \le |\mathscr {C}'|$). Problem (P2) can be solved in the same way.

## Footnotes

1 A path is essentially a connected, finite sequence of straight line segments, and the length of a path is the sum of the Euclidean lengths of each of the line segments.

2 We summarize the argument for those readers familiar with the theory of posets: Consider the *order complex* of the poset $(\mathfrak {T}(X),\preceq )$. The fact that every order complex is a *flag complex* implies, by a classical result of Gromov (1987), that the orthant space is CAT(0).
